# The Reconstruction of Peripheral Auditory Circuit: Recent Advances and Future Challenges

**DOI:** 10.1002/advs.202410494

**Published:** 2025-03-24

**Authors:** Zhe Li, Ying Zhang, Zhenyu Xiao, Jieyu Qi

**Affiliations:** ^1^ School of Life Beijing Institute of Technology Beijing 100081 China

**Keywords:** biomaterials, hearing loss, peripheral auditory circuit, reconstruction, stem cell

## Abstract

The auditory circuit primarily consists of peripheral auditory organs and auditory neural pathways. Hearing loss (HL), as reported by the World Health Organization, affects more than one in eight people worldwide, often leading to severe dysfunction that detrimentally impacts patients' quality of life. Therefore, auditory reconstruction has emerged as a persistent research hotspot and challenge within the biomedical field. Traditional methods for auditory reconstruction, such as drug therapy, hearing aids, cochlear implants (CIs), and so on. To a certain extent, it can help patients with HL improve their hearing status. However, they possess inherent advantages and limitations. Recent advancements in biomaterials, gene editing, stem cells, organoids, and other technologies have ushered in new prospects for the treatment of hearing impairment. This review focuses on the potential of innovative technologies in biomaterials, stem cells, and gene editing in hearing recovery. It reviews the current research status of inner ear hair cell (HC) regeneration, spiral ganglion neuron (SGN) regeneration, and inner ear organoid construction in the auditory loop. Furthermore, the review discusses the challenges associated with these approaches and explores potential future directions, aiming to furnish a comprehensive reference for both research and clinical applications in the domain of peripheral auditory restoration.

## Graphical abstract

This paper summarizes the potential of biomaterials, stem cells, and gene editing technologies in the regeneration of inner ear hair cells, spiral ganglion neurons, and inner ear organoids. Challenges and potential developments are discussed and explored. It aims to provide a comprehensive reference for research and clinical applications in the field of peripheral auditory repair.

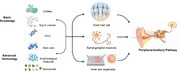

## 1 Introduction

With the advancement of society and the intensification of population aging, HL has emerged as a significant public health concern. With rapid progress in medical technology, the research on the mechanism of HL continues to deepen our understanding of the mechanisms underlying HL, leading to more diverse diagnostic and therapeutic approaches.^[^ ^1^, ^2^ ^]^ In clinical practice, a comprehensive understanding of the auditory circuit is of great significance for the diagnosis and treatment of diseases such as hearing impairment.^[^ ^3^, ^4^ ^]^ Restoring or improving hearing is crucial for maintaining an individual's daily life and development. In addition, reducing the prevalence and severity of HL can significantly improve the productivity and operational efficiency of the entire society.

There are numerous strategies available to intervene and restore sensory and balance functions, including drug therapy, gene therapy, nanotechnology, wearable devices, and assistive technologies.^[^ ^5^, ^6^, ^7^, ^8^, ^9^, ^10^ ^]^ For example, for patients with sensorineural deafness, currently commonly used treatment methods such as hearing aids and CIs have certain effects, but they still have limitations. Some research teams have conducted a number of basic and clinical trial studies on gene therapy for congenital DFNB9 deafness.^[^ ^11^, ^12^, ^13^ ^]^ For the first time in the world, this series of studies publicly published the results of a clinical study of gene therapy for hereditary deafness, showing that AAV vector‐mediated gene replacement therapy is initially safe and can restore hearing to normal levels in patients with severe deafness.^[^ ^12^, ^13^ ^]^ Although these research advances offer new hope for the treatment of HL, the development of therapeutic interventions for HL will still need to be based on human cells and effectively control the signaling pathways of tissue development. Some research focuses on regenerating HCs or auditory neurons from stem cells,^[^ ^14^, ^15^, ^16^ ^]^ and some research focuses on combining with new clinical technology systems such as neural stem cell transplantation and CIs to rebuild the auditory circuit and fundamentally restore hearing.^[^ ^5^, ^17^, ^18^ ^]^ In addition, in vitro models of the inner ear, such as organoids and organ‐on‐a‐chip, can help identify new protective or regenerative drugs, and the development of new gene therapies and stem cell therapies.^[^ ^19^, ^20^, ^21^, ^22^ ^]^ These models hold significant potential for future clinical applications.

Therefore, advances in stem cell technology, biomaterials, and organoid culture can provide unique opportunities to model diseases of the inner ear and develop personalized therapies for HL. However, several challenges must be addressed to achieve their wider and effective applications. In this review, we reviewed the existing biological and physicochemical factors that promote the regeneration of auditory neurons and HCs. It summarizes cutting‐edge work of biomaterials and stem cells used in the construction of inner ear organoids. We also discussed the limitations of existing peripheral auditory loop reconstruction technologies and the prospects. The goal of this review article is to foster progress in medicine and enhance human health.

## 2 Peripheral Auditory Circuit

**Figure 1:**
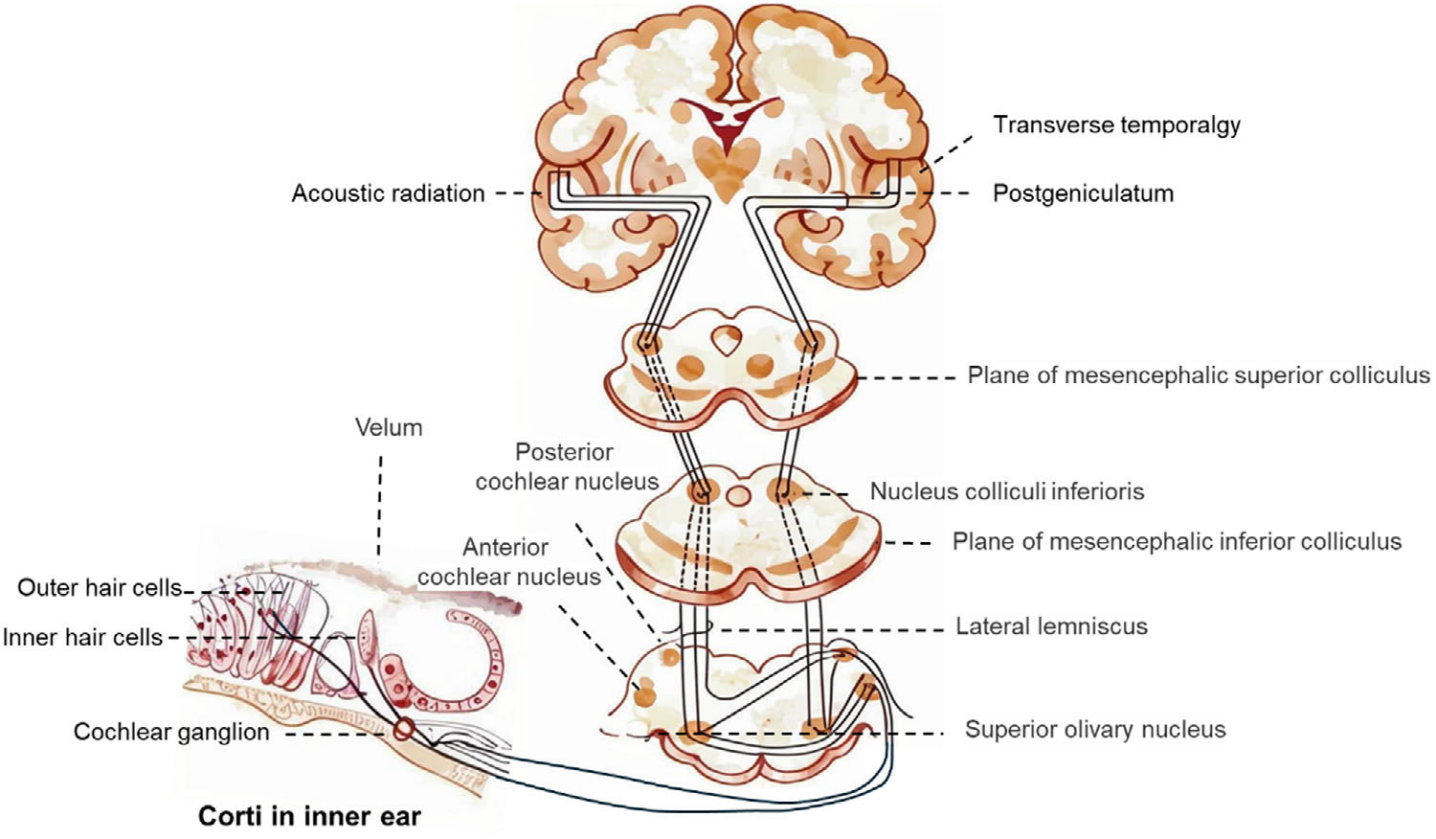
The scheme of the auditory circuit.

The auditory circuit refers to the system composed of the entire path and related neural structure through which sound signals are transmitted from the peripheral auditory organ to the auditory center of the brain for processing and perception (**Figure** **1**).^[^ ^4^, ^23^ ^]^ Sound is transmitted through the outer ear and middle ear to the HCs, auditory nerve endings, and SGNs in the inner ear to obtain hearing. Thereafter, the auditory nerve reaches the auditory cortex through the brain stem conduction pathway. The cerebral cortex center further analyzes, identifies, integrates, and transmits the sound to the corresponding motor center and speech center. Finally, it issues instructions from the center to the effector nerve. That's the entire auditory circuit.

**Figure 2:**
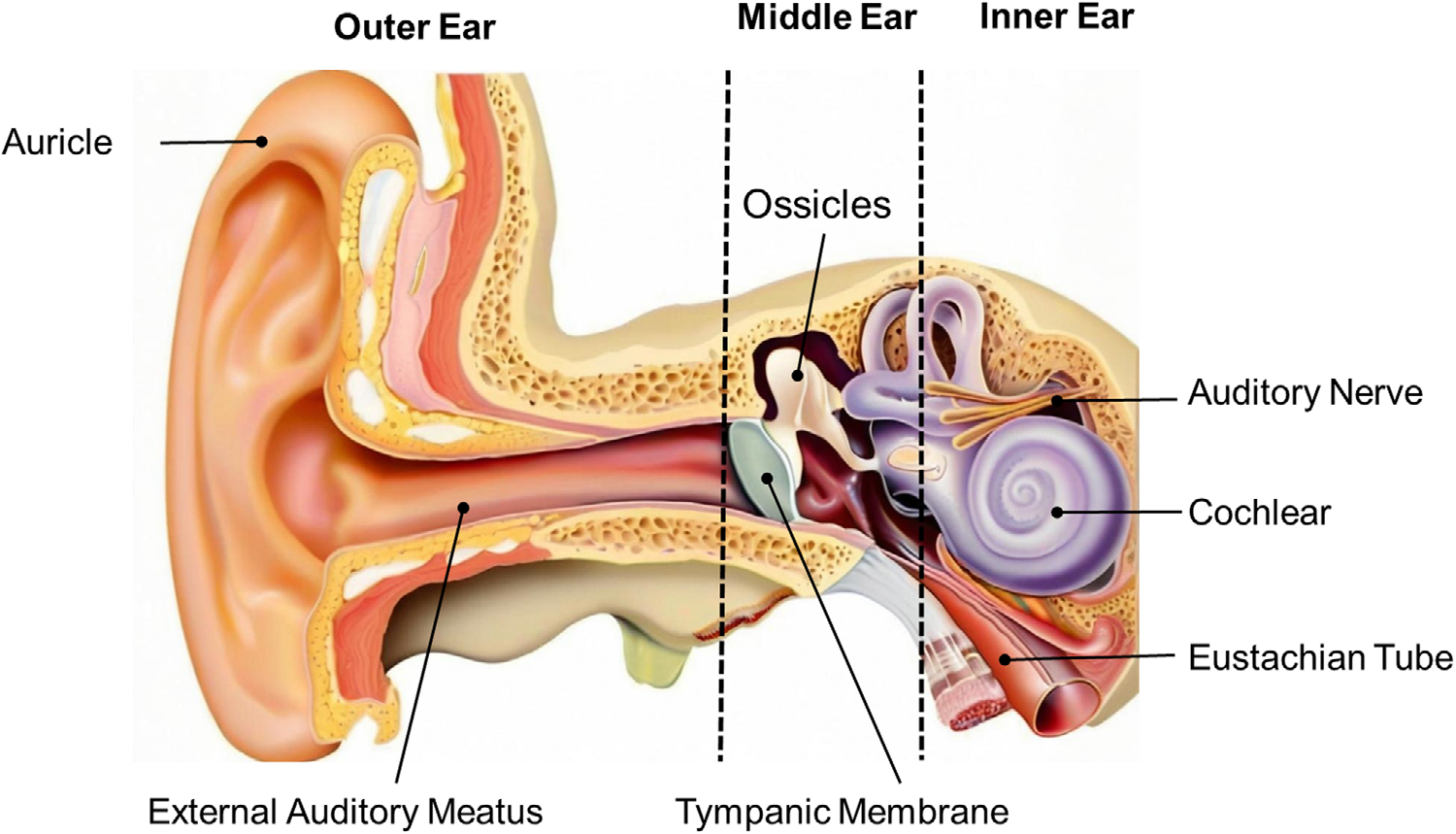
The scheme of the peripheral auditory circuit.

Peripheral auditory circuit is a structure and pathway in the human auditory system that is located outside the brain and is responsible for collecting and initially processing sound signals. It mainly consists of three parts: the outer ear, the middle ear, and the inner ear (**Figure** **2**). The outer ear includes the auricle and the outer auditory canal. The middle ear is located between the outer ear and the inner ear and consists of the tympanum, eustachian tube, tympanic sinus, and mastoid cells. Among them, there are three auditory ossicles in the tympanum that are connected to form the ossicular chain, namely, the malleus, the incus, and the stapes. The inner ear is a key part of hearing and contains structures such as the cochlea. Sound waves are collected by the auricle and transmitted to the outer auditory canal, where they are further transmitted to the eardrum. When sound waves make the tympanic membrane vibrate, the vibration of the tympanic membrane will drive the ossicular chain to vibrate, transmitting the vibration of the sound wave to the inner ear. When the acoustic vibrations transmitted through the middle ear reach the inner ear, they cause the lymph fluid of the inner ear to vibrate. HCs in Corti that are located on the basement membrane of the cochlea bend in response to the vibration of lymph fluid. The movement of the cilia at the top of the HC in the same direction opens ion channels that depolarize the HC, creating an action potential that converts the mechanical energy of sound waves into bioelectricity. These bioelectrical signals will be transmitted to the brain through the auditory nerve composed of axons of spiral ganglion neurons, and finally make the brain produce hearing.

Peripheral auditory circuit is an important path that converts external sound signals into neural signals and transmits them to the brain. Its various parts work together, and any problem in any link may lead to HL. Relevant research teams from all over the world have long focused on the regeneration of HCs through stem cells to repair the structure and function of the cochlea, so as to fundamentally restore hearing.^[^ ^24^, ^25^, ^26^ ^]^ A new clinical technology system combining neural stem cell transplantation and cochlear implantation has also been established. The CI replaces the function of HCs, and the functional auditory neurons are regenerated by neural stem cells, thus rebuilding the peripheral auditory circuit, and restoring the auditory function.^[^ ^27^, ^28^ ^]^

## 3 The Regeneration of Inner Ear HC

The Regeneration of inner ear HC includes the regeneration of stem cell‐derived inner ear HC, the transdifferentiation of supporting cells into HCs and the regulation of new HC maturation.

### 3.1 The Regeneration of Stem Cell‐Derived Inner Ear HC

Embryonic stem cells (ESCs) are the basis of mammalian development and have the potential to differentiate into any type of cell in the organism. Koehler et al. cultured mouse ESCs in a 3D system that was able to induce the differentiation of the inner ear sensory cell epithelia and reproduce HC development.^[^ ^29^ ^]^ The induced HCs develop into hair bundles, which have a similar function to type II vestibular HCs. Co‐culture is also a way to induce cell differentiation. Carpena et al. co‐cultured ESCs with HEI‐OC1 HC lines for 14 days to obtain inner ear progenitor cells and HCs.^[^ ^30^ ^]^

Pluripotent stem cells (PSCs) have similar properties to ESCs, avoiding the ethical issues associated with using ESCs. In 2006, Takahashi and Yamanaka first proposed and demonstrated that the introduction of four transcription factors – Oct3/4, SOX2, c‐Myc, and Klf4‐ can induce the reverse differentiation of mouse fibroblasts into PSCs.^[^ ^31^ ^]^ Oshima et al. obtained inner ear progenitor cells from ESCs and iPSCs through directed induction, and these cells gradually acquired HC‐like traits through the regulation of growth factors.^[^ ^32^ ^]^ However, the accumulation of chromosomal aberrations during the cultivation of PSCs limited the expansion and differentiation of organoids.^[^ ^33^ ^]^ Nie et al. proposed a method to use HCs from human PSCs to gradually regulate BMP, FGF, and WNT through recombinant proteins and small molecules to grow sensory structures in the inner ear.^[^ ^34^ ^]^

In addition, the discovery of several signaling pathways associated with HC regeneration in the inner ear has led to a variety of small molecule compounds regulating HC regeneration capable of regulating this process.^[^ ^35^, ^36^, ^37^, ^38^, ^39^ ^]^

Wnt signaling is necessary for the differentiation of animal HCs.^[^ ^36^ ^]^ Lgr5 is a cell membrane receptor of the Wnt pathway and an important marker of inner ear stem cells. McLean et al. used a small molecule cocktail to achieve a high rate of Lgr5+ cell support cell proliferation and HC differentiation in cochlear organoids.^[^ ^37^ ^]^ They found that the glycogen synthase kinase 3β (GSK3β) inhibitors CHIR99021 and bFGF had the greatest effect on the number and percentage of Lgr5‐GFP progenitor cells. Frequency Therapeutics evaluated the safety and hearing effects of CHER‐911 and VPA (called FX‐322 in their study) by reprogramming progenitor cells to activate the innate HC regeneration potential in humans to restore noise‐induced or sudden sensory nerve‐related HL. Though Cher‐911 and VPA can activate the Wnt signaling pathway and promote the plasticity of adult support cells, regulating the Wnt signaling pathway alone cannot achieve functional HC regeneration for hearing recovery.^[^ ^37^ ^]^ In addition, Kastan et al. found that TRULI is a potent and non‐toxic competitive inhibitor of atp in Lats1/2 in the Hippo signaling pathway that initiates the regeneration of mitotic HCs.^[^ ^35^ ^]^

Another effective method of multigene co‐regulation of inner ear stem cells is to use viral and non‐viral vectors to inject exogenous transgenes into the inner ear through the circular window membrane, semicircular canal, or cochlear stoma.^[^ ^40^ ^]^ Viral vectors used for gene therapy in the clinic usually include recombinant AAV, retrovirus, lentivirus, etc. Due to the AAV‐ie having no effect on the cochlear epithelium or auditory system, it has been used for HC regeneration therapy.^[^ ^41^, ^42^ ^]^ In 2012, Burns et al. used AD‐mediated co‐transduction of Oct3/4, Klf4, Sox2, and c‐Myc with anti‐degradation T58A mutants to induce significant responses in proliferative stimulation and accelerated entry of adult intracellular serphocyte s phase.^[^ ^43^ ^]^ In addition, Lu et al. used Ad‐YAP to induce cultures of cochlear hyperplasia after exposure to neomycin.^[^ ^44^ ^]^ Despite these promising findings, potential immune, and carcinogenic effects have limited their clinical use.

Currently, HC‐like cells obtained from ESCs or iPSCs using prior art have the morphological and electrophysiological characteristics of vestibular HCs, but not those of cochlear HCs.^[^ ^45^ ^]^ Therefore, it is necessary to identify the potential mechanisms of induction of cochlear organoids by ESCs or iPSCs, and to identify the progressive events and key time points of cochlear sensory epithelium development and directed HC differentiation. This knowledge not only contributes to the construction of cochlear organoids but also supports the functional differentiation of adult stem cells into HCs. Furthermore, adult inner ear stem cells are effective resources for in situ repair and regeneration of damaged HCs. However, it is important to note that the plasticity of inner ear stem cells declines rapidly after birth. With clinical applications in mind, it is necessary to develop regulatory approaches to fully restore the plasticity of inner ear stem cells.

### 3.2 The Transdifferentiation of Supporting Cells into HCs

Cell transdifferentiation refers to a process of a differentiated, mature cell type that is transformed into a differentiated, mature cell type. In the auditory system, it is a closely watched field for the transdifferentiation of supporting cells to HCs. Normally, in the inner ear of mammals, HCs cannot regenerate naturally after damage, which can lead to permanent HL. Supporting cells are relatively abundant, and the development of the support cells to the HCs is expected to provide new strategies for HL repair. Wang et al. showed that injury‐activated Lgr5+ supporting cells can regenerate HCs‐like cells through proliferation and direct reverse differentiation, both in culture and in natural cytoplasmic tissues.^[^ ^46^ ^]^ In addition, Lenz et al. utilized organoids that support cell differentiation with Lgr5+ for drug screening, gene silencing, and overexpression.^[^ ^38^ ^]^

During embryonic development, the Wnt signaling pathway plays a key role in ear development, including the formation of HCs. In studies that support cell transdifferentiation into HCs, activation of the Wnt/β‐catenin signaling pathway has been found to promote this transdifferentiation process. During normal inner ear development, Notch signaling pathway regulates the differentiation balance of HCs and supporting cells through lateral inhibition mechanisms. In transdifferentiation studies, inhibition of Notch signaling pathway can remove its inhibitory effect on transdifferentiation of supporting cells into HCs. Wu et al. inhibited Notch signaling with the gamma‐secretase inhibitor DAPT, while activating Wnt signaling with the β‐catenin nuclear translocation agonist QS11.^[^ ^39^ ^]^ Thereby, it preserved Lgr5+ supporting cells and strongly promoted mitosis HC regeneration.

Atoh1 is a key transcription factor that is essential for the development and differentiation of HCs. The up‐regulation of Atoh1 expression plays an important role in the transdifferentiation of supporting cells into HCs. The high expression of Atoh1 in supporting cells by gene transfection and other techniques can induce the transdifferentiation of supporting cells into HCs. In 2005, Izumi et al. first introduced Atoh1 into the inner ear of deaf guinea pigs via adenovirus (Ad) vectors.^[^ ^47^ ^]^ Ad‐Atoh1 could achieve partial hearing recovery and improvement by inducing HC regeneration. Similarly, Chen et al. delivered Ad‐Math1 in combination with Pax2 to Corti organs in newborn mice to promote HC regeneration after neomycin treatment.^[^ ^48^ ^]^ In addition, Menendez et al. used a combination of four transcription factors, Six1, Atoh1, Pou4f3, and Gfi1. They directly transformed mouse embryonic fibroblasts, adult rat fibroblasts, and postpartum support cells. Finally, the HC‐like cells were successfully induced.^[^ ^49^ ^]^

The methods and techniques of inducing transdifferentiation include small molecule compound induction and gene therapy. In the adult cochlea, through AD‐mediated delivery, transient activation of Myc and Notch transforms supporting cells into HCs in response to Atoh1 administration.^[^ ^50^ ^]^ AAV‐ie can effectively transduce cochlear support cells, HCs, and vestibular HCs. It can also improve the transduction rate of Deutscher cells (≈80%) and phalangeal cells (≈90%).

### 3.3 Regulation of New HC Maturation

There were no substantial differences observed between ESCs and iPSCs in their ability to differentiate along the inner ear lineage.^[^ ^51^ ^]^ In addition, there was no substantial difference in the function acquired by the differentiated HC‐like cells between the two kinds of cells. The current response obtained from mechanically stimulated ciliary bundles in regenerated HC‐like cells is similar to that obtained from immature natural HCs. It has a small current, a wide current shift function, a highly variable presence, a measurable fitness rate, and no directional sensitivity.^[^ ^52^ ^]^ Morphological and electrophysiological analyses of regenerated HCs revealed a common signaling pathway that triggers the development of mechanosensitive hair bundles.^[^ ^53^ ^]^ Moreover, additional signals are needed to specify HC subtypes, such as auditory or vestibular HCs, inner or outer HCs, and type I or II HCs.^[^ ^54^ ^]^

DeJonge et al. used mouse ESCs to induce the inner ear and cultured organoids with a morphology similar to the vestibular organs of the inner ear. The generated HCs also had ion channel expression patterns similar to natural vestibular HCs.^[^ ^55^ ^]^ The induced HCs develop into hair bundles, which have a similar function to type II vestibular HCs. Koehler et al. successively regulated TGF, BMP, FGF, and Wnt signaling to induce multiple vesicle‐like structures in a single stem cell assembly.^[^ ^52^ ^]^ Afterward, Liu et al. optimized, characterized, and used the mouse cochlear organoid platform for high‐throughput screening of more than 1000 FDA‐approved small molecule drugs.^[^ ^56^ ^]^ It was found that the tumor therapy drug regorafenib could significantly promote the differentiation of HCs in cochlear organoids. In addition, further studies have shown that Rifenil can also effectively promote the regeneration and maturation of cochlear HCs.

A variant of AAV‐ie and AAV‐IE‐K558R have similar effects on support cell transduction and HC regeneration.^[^ ^57^ ^]^ However, the non‐specificity of the developed AAV to supporting cells limits its application in functional HCs regeneration. Therefore, the development of efficient and specific AAV targeting supporting cells is the key to the study of HC regeneration.

Despite various research demonstrating the effectiveness of various methods for regulating HC regeneration.^[^ ^47^, ^54^, ^55^, ^56^ ^]^ It is important to note that newborn HCs usually do not fully acquire all the morphological and physiological functions of normal cells, such as regular static ciliary structure, normal mechanical transduction function, and mature synaptic connections.

## 4 Regeneration of Spiral Ganglion Neurons

**Figure 3:**
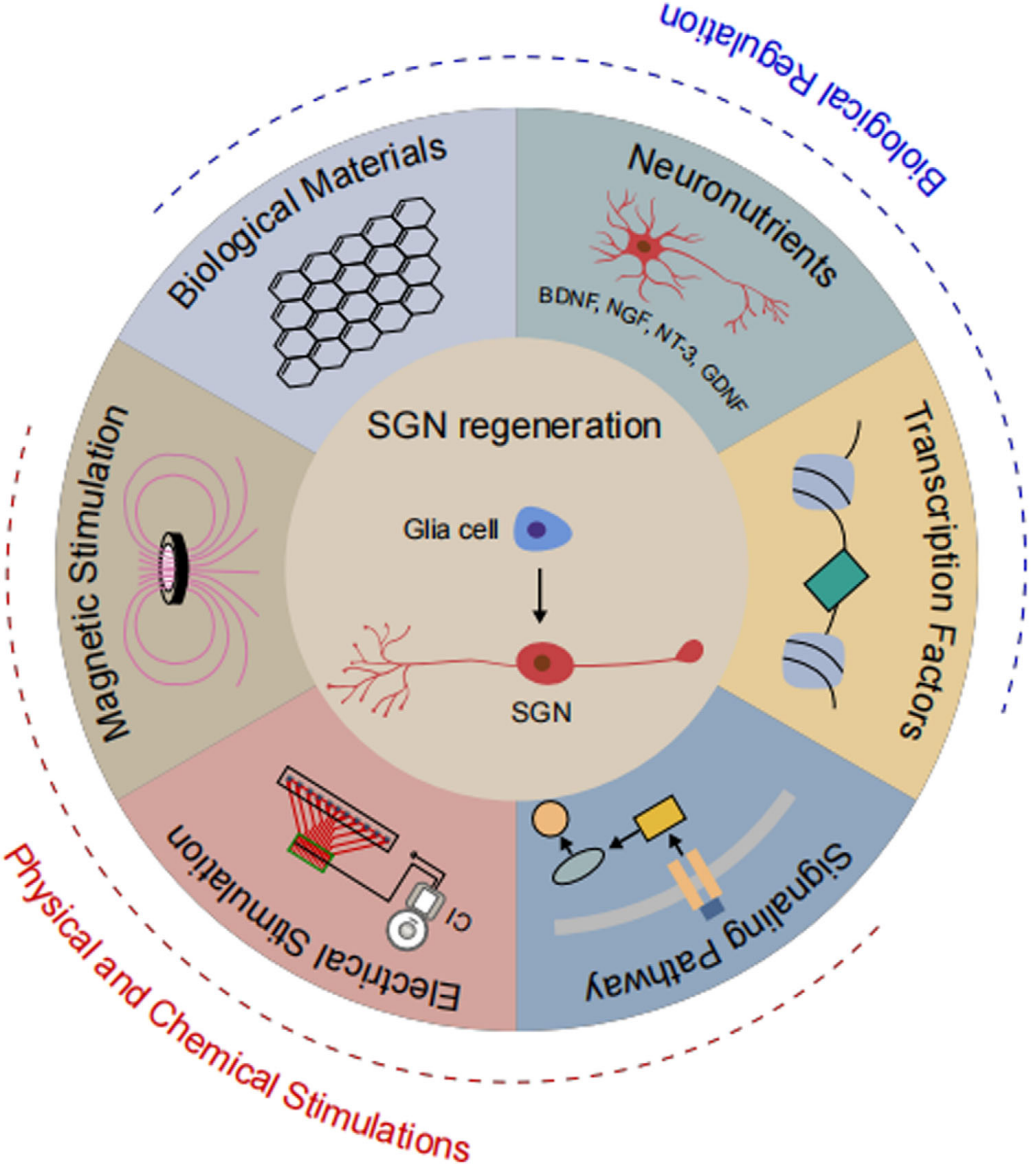
The regulation factors to promote the regeneration of SGNs.

Patients with severe and extremely severe sensorineural HL only have a small number of inner ear HCs and SGNs. CIs have been shown to convert sound into electrical impulses and act directly on SGNs to restore hearing. Although CIs can replace HCs, CI therapy still relies on the remaining functional SGNs. Therefore, it is urgent to find a way to promote the regeneration of SGNs. Regeneration of SGNs involves both axonal regeneration and cell body regeneration. There are reports that neurotrophic factors, electrical stimulation, and biomaterials mainly promote neurite outgrowth, axon plasticity, and some degree of synapse regeneration (**Figure** **3**). In addition, transdifferentiation from glial cells to SGNs and stem cell‐derived regeneration can achieve SGN cell body regeneration. When inducing the differentiation of neurons, the proportion of differentiation into neurons can be increased by adding neurotrophic factors.^[^ ^58^, ^59^ ^]^

### 4.1 Biological Regulation

The main cause of SGN degeneration might be the lack of neurotrophic support from growth factors in sensory HCs and cochlear support cells. In the healthy cochlea, endogenous neurotrophic support ensures the survival of SGN. In both in vitro and in vivo settings, the supply of exogenous neuronutrients can reduce SGN degeneration.^[^ ^60^, ^61^, ^62^, ^63^, ^64^ ^]^ Currently, neurotrophic factors that play a crucial role in the regeneration of spiral neurons from stem cells mainly include: 1) Nerve Growth Factor (NGF), which plays a significant role in the survival, growth, and differentiation of neurons. It can promote the differentiation of stem cells into neurons and enhance the survival and function of spiral neurons. 2) Brain‐derived Neurotrophic Factor (BDNF), which contributes to the development, survival, and synaptic plasticity of neurons. When stem cells regenerate spiral neurons, BDNF can stimulate the growth and branching of neurons, enhancing their functional connections. 3) Neurotrophic Factor‐3 (NT‐3), which is essential for the development and maintenance of sensory neurons, including spiral neurons. It can promote stem cells to differentiate into spiral neurons and support their survival and maturation. 4) Glial cell‐derived Neurotrophic Factor (GDNF), which has protective and growth‐promoting effects on a variety of neurons, including auditory neurons. In the regeneration of spiral neurons, GDNF can improve the survival and differentiation efficiency of stem cells.

Studies have shown that growth factors such as BDNF and NT‐3, produced by cells, can regulate neuronal survival, neuronal differentiation, and axon growth.^[^ ^65^, ^66^ ^]^ Additionally, neurotrophic factors have been shown to stimulate neurite growth in SGN.^[^ ^67^, ^68^, ^69^ ^]^ For example, the long‐term delivery of BDNF by porous silica nanoparticles can improve the survival of spiral ganglion neurons, which is critical for enhancing the electric‐neural interface. Due to the limitations of CIs, the strategy of using synchronous electrical stimulation of CIs to improve the survival and growth of SGN has received extensive attention. Although electrical stimulation itself may induce neurotrophic signaling pathways through depolarization,^[^ ^70^, ^71^, ^72^ ^]^ additional neurotrophic factor treatment effectively increased SGN survival.^[^ ^73^, ^74^, ^75^ ^]^ In Schmidt's study, nanoporous silica nanoparticles (NPSNPs) with a diameter of less than 100 nm were loaded with BDNF to test their efficacy as a long‐term delivery system for neurotrophic factors.^[^ ^76^ ^]^ When the nanoparticle surface was modified with an amino group, the neurotrophic factor was continuously released from the NPSNPs during the 80‐day release period. Cell culture studies of NIH3T3 fibroblasts demonstrated that NPSNPs had good overall cytocompatibility. In vitro SGN experiments showed that the survival rate of SGN was significantly improved in the cell culture containing BDNF nanoparticles compared with the control culture without NPSNPs loading (p < 0.001). Importantly, the amount of BDNF released over a period of up to 39 days also increased the survival rate of SGN. Therefore, NPSNPs carrying BDNF are suitable for the treatment of inner ear diseases and the protection and support of SGN. In Fransson's study, the results showed that four weeks after the implantation of EC devices that release GDNF or BDNF in deaf guinea pigs, SGNS were significantly protected and their electrical reactivity was maintained.^[^ ^77^ ^]^ There is a significant difference between BDNF and GDNF, and GDNF is more favorable. This study suggests that bioprotective substances released by implantable EC devices may provide a viable approach for the treatment of progressive hearing impairment.

While neuroblastocytes are layered, glial cells migrate from the neural crest to serve as supporting cells for SGNs.^[^ ^78^ ^]^ Under normal circumstances, the proliferation and regeneration of glial cells in the inner ear is very weak. Therefore, there is an urgent need to find ways to improve the regeneration of glial cells in SGNs. Neurogenic transcription factors have been widely used in many glial cell reprogramming studies. Several transcription factors involved in neurogenesis and differentiation have been identified, such as Neurog1, NeuroD1, Ascl1, Gata3, Sox2, Sox10, and Prox1. Reyes et al. hypothesized that the addition of pre‐neural transcription factors to neurotrophic factors would increase the number of SGNs formed.^[^ ^79^ ^]^ They used Neurog1, BDNF, and GDNF to obtain an increased proportion of differentiated cells similar to SGN in the auditory nerve. At 5 days in vitro, cells exposed to neurotrophic factors expressing neurog1 showed a denser neural network than cells not exposed to these factors. Notably, the combination of the two factors led to a better reprogramming of active glial cells into SGNs than using a single gene alone. In addition, viral vector‐mediated expression of neurogenic transcription factors makes it more convenient and efficient to reprogram glial cells into SGNs. For example, Chen et al. demonstrated in 2020 that AAV‐mediated ectopic overexpression of NeuroD1 contributes to the conversion of astrocytes to neurons in the adult mammalian brain.^[^ ^5^ ^]^ Nevertheless, efforts are still needed to improve the effectiveness of SGN regeneration, especially to promote the maturation of newborn neurons.

In addition, using stem cells to recover the damaged sensory circuitry is a potential therapeutic strategy. A study noted that the implantation of adult neural stem cells into the damaged inner ear (after chemical deafness) improved survival,^[^ ^80^ ^]^ suggesting that the damaged tissue may release factors that the cells need to survive.^[^ ^81^ ^]^ Hu et al. transplanted neural element 2 (NGN2) transduced adult mouse neural stem cells into deaf guinea pigs and showed that the surviving cells expressed the neuronal marker neuro‐class III β‐tubulin.^[^ ^82^ ^]^ The transplanted cells were close to the sensory epithelium and adjacent to SGN and its surrounding processes, suggesting that adult neural stem cells could survive and differentiate in the damaged inner ear. However, the survival rates remain low, with less than 0.1% of the implanted cells estimated to survive inside the cochlea. Subsequently, Chen et al. induced the differentiation of human‐derived embryonic stem cells (hESCs) into ear epithelial progenitors and ear nerve cells by FGF3 and FGF10, respectively.^[^ ^83^ ^]^ Then, they were transplanted into an auditory neuropathy model of gerbils who were deaf due to Ouabain treatment. It can be seen that otic neuroprogenitors engrafted, differentiated, and significantly improved auditory‐evoked response thresholds. The results could stimulate further research into the development of a cell‐based therapy for deafness.

Although a series of research advances have been made to achieve the protection and differentiation of SGN through biological regulation, there are still some limitations. First, the newly generated SGNs need not only to have neuronal morphology and neuronal protein markers, but also to have complete electrophysiological functions, generate action potentials, and form synaptic connections with conduction neurons in the inner ear HCs and the brain stem.^[^ ^84^ ^]^ Second, the existing research is still in the stage of in vitro cell experiments and animal experiments, and it is necessary to further verify the research results based on more human experiments and finally apply them to the clinic. Third, by binding to corresponding receptors, neurotrophic factors activate intracellular signaling pathways, thereby regulating the fate of stem cells and the regeneration process of spiral neurons. However, the specific mechanism of action and the synergistic relationship between them are still under constant research and exploration. In addition, the lack of SGN‐specific biomarkers has greatly hindered research and clinical applications. Last but not least, there is an urgent need to develop targeted small‐molecule drugs for the effective delivery of biomolecules. In summary, identifying the regulatory factors involved in SGNs regeneration and formulating interventions can promote both the effect and clinical translational potential of auditory nerve regeneration.

### 4.2 Physical and Chemical Factors

#### 4.2.1 Physical Stimulation

Electrical stimulation, as a common physical factor regulating cell behavior, is also effective in regulating the behavior of stem cells. Studies have shown that neural stem cells migrate to the cathode at a higher rate when stimulated by an electric field of physiological intensity.^[^ ^85^ ^]^ The mechanism of action of electrical stimulation on neural stem cells includes regulating the secretion of growth factors, interfering with the cell cycle by increasing the number of cells entering the S phase, increasing the level of intracellular calcium, and activating the proliferation signaling pathway, such as the PI3‐Akt pathway and the ERK1/2 pathway.^[^ ^86^ ^]^ There have also been some reports that low‐frequency sinusoidal electrical stimulation can regulate cytoskeletal recombination and membrane matrix, as well as cell surface receptor redistribution. It may regulate neural stem cell differentiation and neurite growth by altering the ECM protein formulation or intracellular signaling pathways.^[^ ^87^, ^88^ ^]^

Chai's team has designed a CI‐based biocompatible electroacoustic stimulation (EAS) system that delivers electrical stimulation to cells. The effect of the constructed CI/graphene EAS system on SGN behavior was studied.^[^ ^89^ ^]^ The results showed that SGN on the graphene substrate is stimulated by CI transformation, and long‐term electrical stimulation can promote the neurite elongation of SGN. This may be related to promoting the development of growth cones. In addition, Liao et al. combined CI with Ti_3_C_2_T_x_ MXene‐Matrigel hydrogel to establish a 3D electrostimulation culture system.^[^ ^90^ ^]^ The study found that low‐frequency electrical stimulation promotes neurite extension and intercellular signaling in SGN. Guo et al. designed a device capable of electrically stimulating neural stem cells by combining a CI with a graphene substrate.^[^ ^86^ ^]^ Graphene is used both as a substrate for cell culture systems and as a corresponding reference electrode for experiments. In this system, graphene has been shown to significantly promote the neuronal differentiation and maturation of NSCs. The study found that the neurotoxicity of cochlea‐based EAS depends on its unique parameters, such as frequency, amplitude, and duration. These parameters can regulate the survival of NSCs and promote the proliferation and differentiation of neural stem cells into neurons under low‐frequency electrical stimulation. However, the acceleration of neuronal differentiation of neural stem cells by low‐frequency electrical stimulation may be the result of the accumulation of various signals, and the specific mechanism needs to be further studied.

In addition, the effect of CI electrical stimulation on SGN survival was also investigated through in vivo experiments. Martijn et al. investigated whether chronic electrical stimulation patterns (amplitude modulation, high pulse rate) in CI users affected SGN survival, morphology, and function in guinea pigs with HL. It was found that chronic electrical stimulation had no effect on SGN density, perinuclear area, and cell circulation. Moreover, the amplitude of the electrically induced auditory brainstem response threshold and the suprathreshold ear abr were not affected.

Among the various physical stimulus acting on SGN, magnetic stimulation has a very prominent feature. Whether it is a DC magnetic field or an AC magnetic field, it has a strong penetration of biological tissue, so that the effect will not be attenuated by the depth of the target. At the same time, the strategy based on functional magnetic nanoparticles can achieve remote intervention and non‐invasive treatment. This is very attractive in the treatment of diseases of the inner ear. At present, there are few reports on the effect of magnetic stimulation on SGN, but most of them focus on nerve regeneration. Changes in neurite growth and orientation after SGN directly ingested SPION were studied by Hu et al. in vitro.^[^ ^91^ ^]^ Chai's research team studied the magnetic colloidal nanoparticles that were self‐assembled into nanochains under the action of magnetic fields.^[^ ^92^ ^]^ The potential relationship between SGNs and nano chains was investigated. Studies on cell adhesion, the guidance of neurite growth, the influence of growth cone development, and synaptic generation have revealed the potential mechanism of nanochain action on SGNs. These properties enable magnetic colloidal nanochains to become in vitro multifunctional platforms for the study of the directional alignment of SGNs in the future.

#### 4.2.2 Biological Materials

The regulation of stem cells by electrical stimulation also depends to some extent on the microenvironment in which the stem cells grow. The traditional culture system is 2D, such as using porous plates, climbing plates, and petri dishes. Although these basic 2D culture systems are valuable in basic research, they are not suitable for clinical studies that require a large number of cells to be transplanted and regenerated.^[^ ^93^ ^]^ And it is difficult to simulate the internal environment in which cells live in the body. Therefore, it is very important to establish a 3D culture system that simulates the living environment of stem cells in vivo. Cheng et al. reviewed the effects of electrical stimulation with different conductive materials on neural stem cell differentiation.^[^ ^94^ ^]^ The paper suggested that conducting nanomaterials with complex 3D structures, such as conductive hydrogels, carbon nanotubes, and other nanomaterials, are more effective strategies for stem cell therapy. At the same time, because it can provide a cell biophysical environment and is easy to be compatible with various stimuli, it has also become one of the promising methods in vivo applications. Relevant studies have shown that the core mechanism of NSC differentiation into SGN induced by materials is the regulation of signal transduction through protein/RNA and cellular interaction. Due to the limited number of cells induced in vivo, most studies focused on in vitro induction. NGF was first used to maintain the dryness of NSC and proliferation. Then, the electrical stimulation of carbon‐based materials and hydrogel co‐culture were used to promote the anchor‐orientation of NSCs. Afterward, the ion channel dynamics and intracellular ion homeostasis were changed to make NSCs differentiate into neurons and mature. At present, the materials used to regulate the directional differentiation of neural stem cells into spiral neurons mainly include conductive hydrogels, carbon nanotubes, and other nanomaterials (**Table** **1**).

Superarranged Carbon Nanotubes. It has excellent electrical conductivity, mechanical properties, and a unique surface structure. It has been widely used in neurobiology research. Studies have shown that electrospun nanofiber scaffolds can enhance the orientation and differentiation of rat NSC into neurons and glial cells.^[^ ^95^, ^96^, ^97^ ^]^ Hu et al. assembled superarranged carbon nanoscaffolds onto biocompatible methacrylate gelatin hydrogels.^[^ ^98^ ^]^ The composite inherits the topological structure of superarranged carbon nanomaterials and the biocompatibility of hydrogels. Studies have shown that SGNs grown on the composite have higher calcium activity. It can not only promote the transmission of neuronal signals but also promote the growth and maturation of SGNs by promoting the elongation of neurites with cell orientation and the development of growth cones. Therefore, it has great potential in promoting SGNs regeneration. The surface of Morpho Menelaus butterfly wings has a highly anisotropic topology and has been shown to be useful as electronic sensors,^[^ ^99^ ^]^ photonic devices,^[^ ^100^ ^]^ etc. At the same time, this substrate can also be used to regulate cell orientation.^[^ ^101^, ^102^, ^103^ ^]^ Wen et al. constructed the substrate by assembling superarranged carbon nanotubes parallel to the wings of Morpho Menelaus butterflies and covering them with biocompatible methacrylate gelatin hydrogels.^[^ ^103^ ^]^ The superarranged carbon nanotube integrated fins prepared in this study can guide the growth direction of SGNs and promote the growth of SGNs neurites due to their unique 3D topology. At the same time, due to the excellent electrical conductivity of the material, it provides a platform for the research of the electroacoustic stimulation system based on SGNs.

Graphene. Graphene materials have unique physical properties such as high charge mobility and good mechanical strength. It also has good cytocompatibility and biosafety. Therefore, it is widely used in medical fields such as tissue engineering,^[^ ^86^ ^]^ drug and gene delivery,^[^ ^91^ ^]^ and biosensors.^[^ ^92^ ^]^ Tang et al. inoculated NSCs on polystyrene tissue culture plates (TCPS) added with graphene and observed more filopodia, lower resting membrane potential (VR), and more frequent excited action potential (AP) during cell proliferation and differentiation.^[^ ^93^ ^]^ In addition, graphene can increase the expression of TREK‐1 channels in the K2P channel family and enhance the electric field in the microenvironment, promoting synergies between cells. Therefore, graphene can promote cell adhesion and the maturation of the electrophysiological properties of neurons.^[^ ^93^ ^]^

Hydrogel Composite System. The multi‐walled super‐oriented CNT cross‐linked with gelatin methacrylate (GelMA) hydrogel can be used as the neural interface of SGN extracted from the cochlear axis of cultured mouse cochlea.^[^ ^94^ ^]^ The biocompatibility and cell adhesion of CNT are greatly improved. Compared with TCPS, GelMA‐CNT can promote the oriented growth of SGNs, as well as the elongation of neurites and growth cone development. The GelMA‐ACNT‐CI electrical stimulation system was constructed, and through immunofluorescence and calcium imaging, it was found that low‐frequency electrical stimulation can promote neurite elongation. The material can increase calcium activity, promote synaptic signal transmission, and functional maturation of SGN.

MXene Nanomaterials and Their Derivatives. The general formula for MXene is Mn+1XnTx (n ranges from 1 to 4), where M is the transition metal, X is carbon or nitrogen, and Tx is the abundant functional group at the surface end of the outer transition metal layer.^[^ ^104^ ^]^ Its thickness is only a single atom or a few atoms thick, while its lateral size can be up to a few microns or even larger, depending on how the material is prepared. MXene can be synthesized by the top‐down strategy or the bottom‐up strategy, selectively stripping the A element layer from the precursor to transform it into the MAX phase or the non‐Max phase, which is a typical top‐down method. However, the bottom‐up method is not suitable for biomedical engineering due to its shortcomings such as low yield and lack of surface modification ability.^[^ ^105^ ^]^ MXene and its derivatives have attracted much attention in biomedical fields, such as regenerative medicine,^[^ ^106^ ^]^ biosensing,^[^ ^107^ ^]^ drug delivery,^[^ ^108^ ^]^ etc. Such materials have also been widely used to construct nerve cell microenvironments. Liao et al. dispersed the surface‐modified Ti_3_C_2_T_x_ MXene thin film on the TCPS. It was found that MXene can promote the proliferation of NSCs and is more conducive to neuronal differentiation and synaptic growth than TCPS. Then, coupled with electrical stimulation (ES), NSCs can promote the maturation of neural networks.^[^ ^90^ ^]^ Furthermore, Li et al. added MXene to the TCPS substrate.^[^ ^109^ ^]^ First, laminin was treated with surface adhesion to promote the anchoring of NSCs. After immunofluorescence staining, it was found that MXene could promote the proportion of NSCs differentiated into neurons and the elongation of neurites. Finally, the electrophysiological characteristics were recorded by patch clamp. It was proved that MXene can increase the voltage‐gated calcium channel current (ICa) of newborn neurons, increase the release of action potential, increase the synaptic transmission between neurons, and promote the electrophysiological development and maturation of neurons.

**Table 1:** Biological Materials used for the regeneration of SGNs.

Materials	Properties	Advantages in neurobiological applications	Refs.
Superarranged Carbon Nanotubes	Highly ordered structure, excellent mechanical properties, good electrical properties, excellent thermal properties and large specific surface area	Excellent electrical conductivity and mechanical properties, and a unique surface structure	[95, 96, 97, 103]
Graphene	Ultra‐thin 2D structure and honeycomb lattice structure, large specific surface area, high strength, high elasticity, high conductivity, special electronic properties, long spin life, high thermal conductivity, good light transmittance, high nonlinear refractive index, high chemical stability and adjustable reaction activity	High charge mobility, good mechanical strength, great cytocompatibility and biosafety	[86, 91, 92, 93]
Hydrogel Composite System	High water content, flexibility and elasticity, good biocompatibility, controllable physical and chemical properties, good adsorption and load capacity, mechanical properties can be enhanced, and response to external stimuli	Good biocompatibility, flexibility	[94, 98]
MXene Nanomaterials and Their Derivatives	Large specific surface area, more active sites, good electrical conductivity, high mechanical properties, rich chemical activity, adjustable band gap and excellent hydrophilic properties	More conducive, excellent hydrophilic properties	[90, 109]

Stem cell differentiation is a complex process regulated by numerous external and internal factors. However, due to the complexity of stem cell differentiation, the outcome of this processes is influenced by the type of cells and the conditions of embryonic stem cells involved.^[^ ^94^ ^]^ In conclusion, biological factors and physicochemical factors promote stem cell regeneration, and SGNs hold great promise in stem cell therapy. In the future, more research should be conducted on the mechanism of 3D scaffolds made of biological materials and electrical stimulation to promote stem cell differentiation. The combination of new conductive materials with stem holds considerable promise for advancing the application of stem cell therapy in HL and even the treatment of neurological diseases.

## 5 Inner Ear Organoids

Inner Ear organoids are cells that are cultured in vitro through specific techniques and conditions and have structural and functional characteristics similar to those of cochlear in vivo. First, the stem cells are inoculated on a specific culture dish or scaffold. Then, by adding a series of growth factors, cytokines and chemicals, the microenvironment of cochlear development in vivo was simulated. It could induce stem cells to differentiate and develop in the direction of cochlear cells, and eventually inner ear organoids with certain structure and function were formed. With the construction of inner ear organoids, it is helpful to study the developmental process of cochlea and the mechanism of physiological and pathological changes. In addition, it can provide an important platform for drug screening, regeneration of HCs and SGNs. Finally, it can provide new ideas and methods for the repair of peripheral auditory circuits.

### 5.1 Cell Source

Inner ear organoids can be induced from pluripotent stem cells or unipotent stem cells, including embryonic stem cells, induced pluripotent stem cells, and inner ear stem cells.

#### 5.1.1 Embryonic Stem Cells

The inner ear originates from the base plate of the ear and is located at the otico‐epibranchial placode domain (OEPD).^[^ ^110^, ^111^ ^]^ The upper cortex, neuronal cells, and glial cells of the inner ear are derived from ectodermal cells, while the mesenchymal cells surrounding the inner ear are derived from the mesodermal layer and the cranial neural crest.^[^ ^112^ ^]^ The differentiation of human embryonic stem cells (human ESCs) into specific tissues requires the simulation of complex pathways and regulatory networks composed of transcription factors during embryonic development.

Ding et al. used cochlear epithelial progenitor cells (OEPs) induced by human ESCs to differentiate in chicken elliptical cyst stromal cell conditioned medium supplemented with EGF and retinoic acid.^[^ ^113^ ^]^ Clusters of epithelial cells similar to HC‐like cells were formed, and the cells showed ciliary tracts composed of actin. In vitro culture of human ESCs can provide assistance for the study of signaling pathways. In addition, Chen et al. used the human ESCs cell line X1 to isolate cell clusters and cultured them with N2, B27, FGF3, and FGF10 for 12 days to successfully obtain differentiated inner ear progenitor cells.^[^ ^114^ ^]^ The temporal expression pattern of Notch ligand and receptor, a key signal regulating HC differentiation, was detected by quantitative reverse transcriptase‐polymerase chain reaction, suggesting that JAG2 and DLL1 may have a synergistic effect in the process of HC differentiation in vitro.

#### 5.1.2 Induced Pluripotent Stem Cells

Compared with ESCs, iPSCs have similar differentiation pluripotency and self‐renewal ability. In terms of source, iPSCs can be induced by somatic cells in vitro, avoiding the use of embryos in the past and thus reducing the controversy of medical ethics.^[^ ^115^ ^]^ At the same time, iPSCs are easy to obtain and can be cloned in large numbers, providing a rich source of cells for stem cell research and cell therapy in vitro.

Studies have shown that iPSCs can induce differentiation into inner ear HCs. So far, almost all studies of iPSCs induction of inner ear organoids have been improved on the basis of the step‐by‐step induction method established by Karl R Koehler et al.^[^ ^17^, ^115^ ^]^ Most of the induced HCs showed the characteristics of type II vestibular HCs. The induction steps mainly include: 1) the formation of epidermal ectoderm and ear substrate, 2) the formation of inner ear progenitor cells, and 3) the formation of intracavitary fine structures with sensory epithelium.^[^ ^116^ ^]^ For example, Tang et al. demonstrated the feasibility of creating organoids from disease models through the genetic manipulation of iPSC‐derived organoids from deaf mice with the Tmprss3Y260X mutation.^[^ ^117^ ^]^

#### 5.1.3 Inner Ear Stem Cells

The establishment of in vitro neurosphere culture methods has facilitated the development of in vitro ear cell mass culture techniques, which facilitates the separation of putative stem cells or progenitor cells remaining in the sensory tissue of the inner ear.^[^ ^118^, ^119^ ^]^ Although the source of the clonogenesis, self‐renewal, and pluripotency of the inner ear cell mass has not been investigated, studies have identified progenitor cells in the sensory epithelium of the cochlea that can differentiate into supporting cells or HCs in vitro^[^ ^120^, ^121^, ^122^ ^]^ and SGN isolated populations of cells can differentiate into sensory neurons. Over the years, studies in chickens, rodents, and other species have shown that there are two mechanisms for endogenous regeneration of inner ear HCs: direct transdifferentiation of supporting cells into HCs, and mitosis of supporting cells into daughter cells and subsequent differentiation into HCs.^[^ ^123^, ^124^ ^]^ Chai et al. isolated cochlear support cells based on the expression of Sox2, p27, p75, and Lgr5 by cell sorting.^[^ ^125^ ^]^ The results showed that several subtypes of supporting cells had proliferation ability.

The Wnt signaling pathway plays a key role in the development and formation of the cochlea, among which Lgr5 is considered to be one of the most rigorous stem cell marker genes in mouse cochlea progenitor cells.^[^ ^125^, ^126^, ^127^ ^]^ HCsA study showed that Lgr5‐positive cells in the cochlea of newborn mice were able to proliferate and differentiate into HCs in response to damage.^[^ ^128^ ^]^ Lgr6‐positive cells are similar to Lgr5‐positive stem cells in that they produce HCs in vitro, and the same number of Lgr6‐positive cells produce more HCs.^[^ ^129^, ^130^ ^]^ Therefore, targeting Lgr6‐positive cells to regenerate HCs may be more efficient, helping to replenish damaged HCs.

Axin2 is a negative feedback gene downstream of the Wnt signaling pathway and is differentially expressed in and around the developing cochlear canal.^[^ ^131^ ^]^ Jan et al. found that Axin2‐positive boundary cells have the characteristics of cochlear progenitor cells and can proliferate into cell colonies and differentiate into supporting cells and HCs.^[^ ^132^ ^]^ In addition, Frizzled9‐positive sertoli cells regenerate into HCs both in vivo and in vitro and have similar proliferation and differentiation capabilities to Lgr5‐positive progenitors.^[^ ^133^ ^]^

Significant progress has been made in the study of stem cells directed to differentiate and form inner ear organoids, but researchers still face many challenges. First, the efficiency and predictability of stem cell differentiation needs to be improved, which will help improve its clinical applicability. Second, products derived from allogeneic stem cells may trigger an immune response and carry the risk of long‐term toxicity or carcinogenicity. Therefore, the issue of immune rejection and safety should be paid attention to. In addition, the use of ESCs and iPSCs is subject to ethical and legal disputes and restrictions. Optimizing production processes, controlling costs, and ensuring quality will help ensure mass production and application. Finally, the long‐term in vivo stability and functional efficacy of the differentiated cells need to be monitored to ensure lasting and safe therapeutic outcomes. Despite these challenges, the results of research on inner ear organoids are remarkable. With the continuous progress of science and technology, it is believed that these challenges will be gradually overcome, providing new prospects for the construction of auditory circuits.

### 5.2 Construction Method

Organoid construction techniques encompass 2D culture and 3D culture. Compared to 2D culture, 3D culture of organoids is closer to living organs in terms of anatomy and function. They form 3D tissue‐like structures through the self‐organization, self‐renewal, and differentiation capabilities of stem cells. This culture technique can better simulate the cellular interactions in vivo, the influence of the extracellular matrix, and the spatial distribution of cells. Additionally, technology also aids in simulating the complex structure and function of organs, providing a more valuable model for drug screening, efficacy evaluation, and the study of disease development mechanisms. In 2014, Koehler et al. first described a culture system utilizing 3D mouse embryonic stem cell culture to generate inner ear organoids, providing a reproducible and scalable method for generating inner ear sensory tissue in vitro.^[^ ^29^ ^]^ Afterwards, Mills et al. in 2016 employed a 3D culture method to generate inner ear sensory cell epithelium, simultaneously generating sensory HCs and neuronal populations.^[^ ^134^ ^]^

There are a variety of methods for 3D culture of organoids, including scaffold‐based culture, stentless culture, and bioprinting technology. Scaffold‐based cultures typically employ natural or synthetic materials as a supporting framework for cell growth, such as collagen, hydrogels, and the like (**Table** **2**).

Matrigel is a commonly used organoid 3D culture scaffold system. The main components of Matrigel are ECM proteins (i.e., 60% laminin, 30% collagen IV, and 8% endomucin) and several growth factors, including insulin‐like growth factor I (IGF‐1), TGFβ, and vascular endothelial growth factor (VEGF). Under the condition of Matrigel culture, isolated inner ear stem cells can be effectively differentiated into inner ear organoids.^[^ ^135^ ^]^ However, due to differences in biochemical characteristics between batches, inconsistent growth factor concentrations, and difficulties in customization, Matrigel exhibits poor repeatability in cell culture experiments and cannot fully match the culture of organoids in different tissues. Thus, by combining with other materials that are well defined chemically and mechanically, synthetic organoid culture scaffolds are studied.

By introducing the Ti_3_C_2_T_x_ MXene nanomaterial into Matrigel, researchers effectively regulated the performance of Matrigel and promoted the development of cochlear organoids.^[^ ^136^ ^]^ This is conducive to the formation of organoid HCs, can improve the efficiency of synaptic formation in the co‐culture system, and further advance the research on organoid development and HL treatment. Although there have been several studies of inner ear organoids based on Matrigel and its composite hydrogels, no other hydrogel materials have been applied to inner ear organoids. Therefore, it is urgent to utilize existing organoid culture materials or develop new hydrogel‐based extracellular scaffolds to promote the application of inner ear stem cells in regenerative medicine.^[^ ^137^ ^]^ For example, polyethylene glycol (PEG) hydrogel is used as the main body for the culture of inner ear stem cells and the construction of inner ear organoids. In addition, based on the suitable biological characteristics and conditioning physical properties of GelMA hydrogels, 3D culture of inner ear organoids is realized by combining GelMA with nanoparticles or other polymers.^[^ ^138^ ^]^

In addition to the modification of hydrogel culture materials, the introduction of specific mediators or cytokines can also effectively enhance the culture efficiency of inner ear organoids.^[^ ^139^ ^]^ A study showed that by precise temporal control of BMP, TGFβ, and FGF signals, the HCs derived from the ESCs aggregation embryoid successively exhibited the functional properties of natural mechanosensitive HCs. At the same time, synaptic connections are formed with sensory neurons derived from ESCs cultures.^[^ ^17^, ^29^ ^]^ Some other research proved that subsequent addition of the gamma secretase inhibitor LY411575 can effectively induce the formation of inner ear organoids.^[^ ^37^, ^140^ ^]^ Cochlear epithelial cells can also be directly isolated from neonatal mice and 3D cultured by adding growth factors such as mitogen under Matrigel culture conditions.^[^ ^141^ ^]^ In addition, studies have shown that the carefully timed treatment with the powerful Wnt agonist CHIR99021 could promote the induction of ear vesicles, a process previously self‐organized by unknown mechanisms.^[^ ^142^ ^]^

Bioprinting can precisely control the distribution of cells and biomaterials to create organoids with specific cell levels and organizational structures, closer to the complex structure of real organs. In addition, bioprinting enables the co‐culture of multiple cell types. However, bioprinting also faces some challenges in organoid construction. For example, the cell survival rate in the printing process, the biocompatibility of biological materials, and the printing accuracy need to be improved. In addition, the nutrients and oxygen supply in the culture process, the monitoring and analysis of cells, and other aspects need to be further improved and optimized. In the future, with the continuous development of technology, bioprinting is expected to make greater breakthroughs in the field of inner ear organoids, bringing more possibilities for disease research, drug screening, and regenerative medicine.

**Table 2:** Methods for the 3D culture of organoids.

Materials	Cells	Advantages	Reported cases	Refs.
BMP4 +SB‐431542+FGF2 +LDN‐193189 + fresh media	ESCs	The ESC aggregates can be transformed sequentially with the precise time control of the signal path	Stem‐cell‐derived HCs exhibit functional properties of native mechanosensitive HCs and form specialized synapses with sensory neurons that have also arisen from ESCs in the culture	[17]
Matrigel or GelMA	ESCs	High culture efficiency	ES cells were directed to differentiate into ear vesicle‐like structures containing functional mature sensory HCs	[19]
BMP, SB, BMP/SB, BMP/SBFGF, BMP/SB‐LDN and BMP/SB‐FGF/LDN	ESCs	Reproducible and scalable, the differentiating cells are free to self‐organize into epithelia	providing a reproducible and scalable method for generating inner ear sensory tissue in vitro	[29]
Ti_3_C_2_T_x_ Mxene‐Matrigel	Primary cochlea cells	Mechanical adjustability and satisfactory biocompatibility	The regeneration and ripening of the organ HCs were promoted	[136]
Rapamycin + Marigel hydrogel	Cochlear epithelial cells	Supporting cell plasticity in an mTORC1‐dependent manner can be promoted by LIN28B	Mouse cochlear epithelial cells can re‐enter the cell cycle, produce HCs, and differentiate into organoids	[141]
Potent Wnt agonist CHIR99021	ESCs and iPSC	The number and the size of otic vesicles derived in 3D culture were increased	Modulation of Wnt Signaling Enhances Inner Ear Organoid Development in 3D Culture	[142]
PPy‐PDA‐Marigel hydrogel	Inner ear progenitor cells	Superior biocompatibility and electrical property	Construct a high‐throughput inner ear organ chip for cochlear organoid culture and drug screening	[143]

In summary, despite significant progress in organoid construction research, numerous deficiencies and challenges remain to be addressed. Further research and methodological innovation are essential to tackle these obstacles and advancing the field.

### 5.3 Applications

#### 5.3.1 Drug Screening

Drug screening for ototoxic, protective, or regenerative compounds is typically conducted in vitro through high‐throughput screening. Organoids derived from specific progenitor cells of mouse cochlear tissue provide a novel tool for studying toxicity and regeneration.

Will J. McLean et al. utilized organoids to screen for combinations of small molecule compounds that are highly effective in inducing HC regeneration.^[^ ^37^ ^]^ The Wnt pathway activator CHIR99021 and the histone deacetylase inhibitor VPA can significantly promote the expansion of inner ear organoids. Additionally, it is noteworthy that CPD3, as a new GSI, can replenish the number of HCs lost in ototoxicity tests after the treatment of cochlear organoids.^[^ ^144^ ^]^

Growth factors, such as EFG, IGH, and FGF, are also required for the culture of inner ear organoids, among which bFGF and CHIR99021 are the most crucial for Lgr5 + cell culture. HCsFor example, ERBB2, a member of the EGF receptor family, is a known regulator of cell cycle progression in different tissues. Activation of the ERBB pathway by chemical or genetic means can induce the proliferation of cochlear support cells and the differentiation of HCs.^[^ ^145^ ^]^ In the inner ear organoids, the combination of ERBB3‐binding protein inhibitors WS3 and WS6 with CHIR99021 significantly promoted the formation of Lgr5‐positive inner ear organoids, but did not affect the differentiation of HCs.^[^ ^38^ ^]^ HCsOn this basis, in order to discover new signals that promote HC differentiation, Liu et al. used the second‐generation cochlear organoids from Pou4f3 (EGFP/+) mice to screen 1004 small molecules approved by the US Food and Drug Administration in the presence of CHIR99021 and the absence of LY411575.^[^ ^56^ ^]^ HCsIn addition, similar to LY411575, regofenil treatment also proved that it can promote the formation of induced presynaptic bands in HCs.

Therefore, the establishment of a high‐throughput cochlear organoid screening platform is of great significance for identifying new small‐molecule drug candidates for promoting HC differentiation and maturation. However, it is worth noting that organoid culture models have certain limitations in drug screening. Since the composition of the medium is chosen to maximize the properties of the stem cells, the results obtained under in vitro conditions may not accurately reflect the changes in an intact organ caused by a single drug in the body. In addition, due to the fact that the supporting cells from the entire cochlea are isolated, assembled, and cultured under homogeneous conditions, cochlear organoids do not have access to precise cell type and anatomical information on damaged or regenerated cells.

#### 5.3.2 Mechanism Research

Inner ear organoids can provide in vitro models for studying HL caused by genetic defects. TMPRSS3 is a type II transmembrane serine protease that is essential for normal hearing in mammals. Mutations can cause congenital and early‐onset HL. The inner ear organoids containing the TMPRSS3 mutation (TMPRSS3Y260X) were able to undergo normal early development, but degradation and apoptosis of HCs were observed in the TMPRSS3Y260X inner ear organoids on day 38 of culture. At the same time, HC BK ion channels were also reduced in the inner ear organoids knocked out by Tmprss3, which was consistent with the apoptosis of mouse vestibular sensory epithelial cells in vivo. This suggests that the inner ear organoids derived from mouse embryonic stem cells can mimic the pathological features associated with genetic mutations in vivo. The results of RNA‐SEQ indicated that the inner ear organoids can be used as a tool to explore the molecular mechanism of HC degeneration caused by Tmprss3 knockout.^[^ ^117^ ^]^ The ability of inner ear organoids containing specific genetic mutations to reproduce genetically related phenotypes observed in vivo suggests that organoids can serve as powerful tools for understanding the genetic basis of mammalian diseases.

GJB2 (gap junction beta 2, GJB2) encodes the gap junction protein 26 (Connexin26, CX26), whose mutation causes severe nonsyndromal sensorineural HL in humans. Due to anatomical limitations, human cochlear cells are not easily biopsied. Therefore, ESCs/iPSCs are an important tool for studying the molecular mechanisms of inner ear pathology and for generating cells for alternative therapies. In order to establish an in vitro disease model for GJB2‐related HL, Fukunaga et al. induced the differentiation of mouse iPSCs into CX26‐expressing cells and formed a typical CX26‐gap junction plaque in cochlea tissue (CX26‐gap junction plaque, CX26‐GJP).^[^ ^146^ ^]^ These CX26‐expressing cells induced by iPSCs exhibit spontaneous Ca2+ transients typical of the developing cochlea, and their Ca2+ activity requires the involvement of ATP and hemichannels, consistent with the normal developing cochlea. In addition, differentiated cells of iPSCs from CX26‐deficient mice also have the potential to form GJP. After inducing GJB2 knockout, small vesicle‐like GJPs with rapid intracellular disruption are the main pathological features of GJB2‐associated HL. This in vitro organoid model facilitates inner ear cell therapy and drug screening for GJB2‐associated HL. The inner ear organoids provide a convenient in vitro disease model and provide a practical basis for the investigation of relevant mechanisms in the inner ear.

iPSCs‐derived inner ear organoid models have also been used to detect the effect of gene correction and study the molecular mechanism of pathology. The MYO7A and MYO15A mutations are known genes for deafness in humans. Chen et al. obtained fibroblasts from patients with MYO7A and MYO15A mutations, cultured abnormal stereocilia in vitro, and demonstrated that the derived HCs recovered the stereocilia‐like projection structure after correcting the gene using CRISPR/Cas9 technology.^[^ ^147^ ^]^ In the study of Hosoya, iPSCs of Pendred syndrome patients with biallelic SLA36A4 locus mutation were extracted to establish a model, revealing the molecular mechanism of HL caused by Pendred protein dysfunction.^[^ ^148^ ^]^

#### 5.3.3 Organ‐on‐a Chip

Microfluidic chips are a technology platform for controlling fluid in the micro‐scale space. Micromachining technologies such as lithography, etching, and polishing enable the fine processing of complex fluid channels and reaction zones, as well as the multi‐functional integration of sample pretreatment, reaction, separation, and detection. It has many remarkable advantages, such as small size, high integration, and precise control. Microfluidic chips are widely used in many fields, such as biomedicine, chemical analysis, and drug screening. For example, in the biomedical field, when used for cell analysis, only a small number of cell samples can be detected. Using microfluidic chips, concentration gradients can be constructed to quickly and efficiently obtain the minimum effective concentration of a drug. In chemical analysis, it is possible to achieve an integrated operation from sample injection to final result output. As a cutting‐edge technology, this technology has brought great potential and innovation space for scientific research and practical application.

Organ on a chip derived from microfluidic chips can highly simulate the complex physiological functions of organs and shows broad application prospects in development research, disease simulation, and new drug research and development. However, less attention has been paid to effectively building CIs on a chip. Hu et al. proposed a novel integrated conductive hydrogel biological hybrid system for CI organoids.^[^ ^143^ ^]^ By combining cochlear electroacoustic stimulation (EAS), the inner ear progenitor cells can proliferate and spontaneously form spheres, and eventually enable the construction of CI organoids. By combining the hybrid system with microfluidic chip technology, dynamic, and high‐throughput evaluation of drugs associated with inner ear diseases can be achieved. These results indicate that the chip cochlear platform has great application potential in organoid culture and deafness drug evaluation.

Therefore, auditory organoids and organs on a chip have broad application prospects in the future. With the deepening of research, it is expected to bring fundamental changes to the treatment of sensorineural deafness and other diseases. On the one hand, by further optimizing the organoid model, it can more accurately simulate the disease state, provide a more reliable experimental platform for drug research and development, and accelerate the market process of new drugs. On the other hand, based on the in‐depth understanding of the mechanisms of organoid development, innovative therapeutic strategies are expected to be developed, such as the use of cell regeneration technology to repair damaged auditory nerves. In addition, the research results of auditory organoids and organs on a chip may be extended to other related fields, such as the treatment of ear diseases such as tinnitus and vertigo. It will bring new hope for improving the quality of life of patients.

## 6 Summary

Hearing is one of the important senses and has significant research and clinical prospects. The auditory circuit represents a highly intricate and delicate system, and in‐depth study of which is essential for a comprehensive understanding of the mechanisms underlying human auditory perception, processing, and sound production. By exploring the mystery of the auditory circuit, we can further reveal the root cause of hearing impairment and provide a theoretical basis for developing more effective treatment methods. This, in return, contributes to advancements in the fields of neuroscience and biomedical engineering and others, offering valuable insights, references, and inspiration for the treatment of related diseases. In this review article, we systematically describe specific methods for regulating the plasticity of inner ear stem cells and the maturation of new HCs. We also examine the effects of biological factors and physicochemical factors on the regeneration of spiral neurons. In addition, we provide a comprehensive analysis of the cell sources, construction methodologies, and potential applications of inner ear organoids, highlighting their prospective role in future auditory system reconstruction which will play an important role in the future treatment of hearing reconstruction. To further advance the protection and restoration of the auditory system, this review summarizes the latest research progress in inner ear HC regeneration, spiral neuron regeneration, and inner ear organoid construction.

Despite significant potential to greatly advance the field of auditory reconstruction, current research remains in its early stages. For example, some experimental drugs and gene therapy approaches have shown potential to promote hearing HC regeneration in animal models, but these approaches are still stuck in the laboratory stage and require further research and clinical trials. The heterogeneity of some 3D culture systems also poses limitations, particularly in their direct application in stem cell‐directed differentiation and organoid construction. In addition, the application of organ repair of cochlear organoids in animal models is still restricted to the in vitro research stage, with no successful cases of in vivo organ repair and functional reconstruction reported to date. In the future, further modification of cooperative regulatory strategies is needed to develop new methods of induction and differentiation of cochlear cell types, including how cochlea‐type HC‐like cells are derived, whether new HCs have cell‐to‐cell connections, whether inner ear organoids are susceptible to the same external damage that causes deafness in humans and other mammals, and how to reproduce in vitro the vasculature that regulates the blood supply to the inner ear.

**Figure 4:**
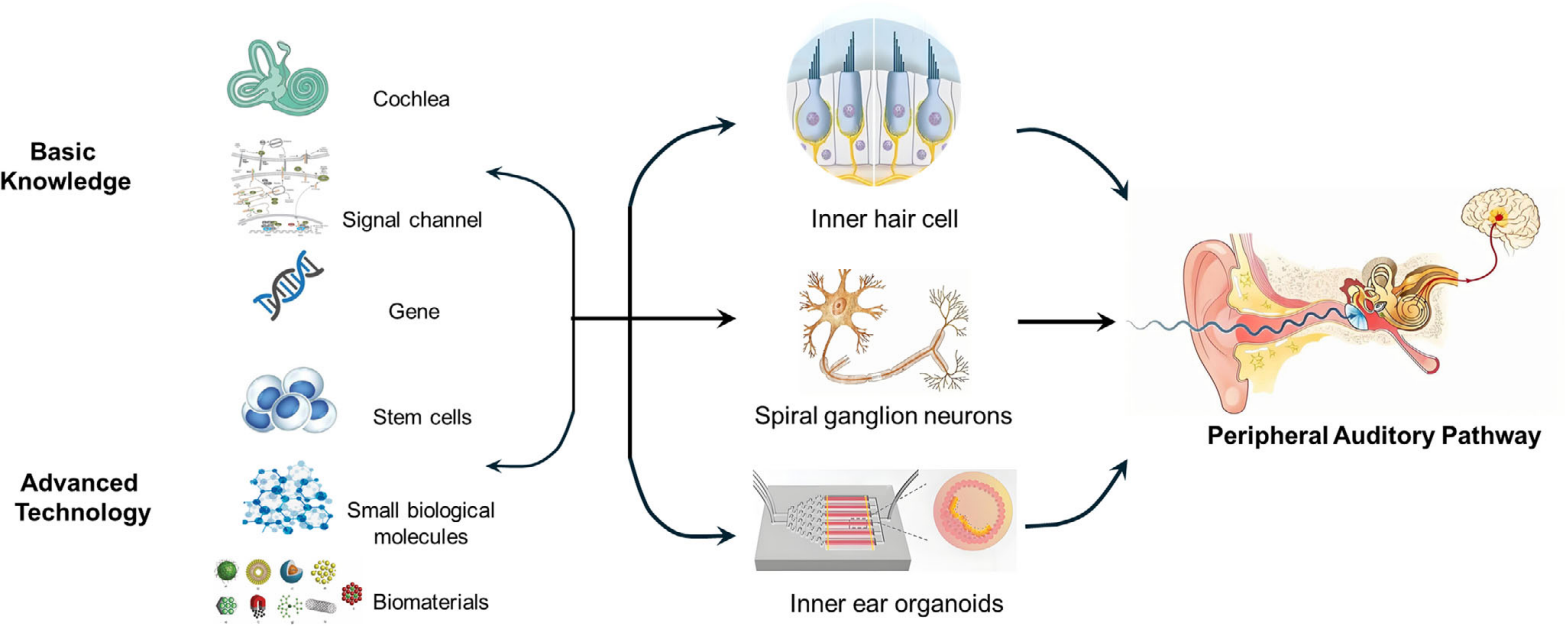
A roadmap to build a more efficient and complete peripheral auditory circuit.

Addressing these challenges requires an in‐depth understanding of the complex anatomy of the cochlea, including the spatial organization of its cellular components (**Figure** **4**). This is fundamental to the successful design and implementation of organizational engineering strategies. Moreover, uncovering the molecular basis of temporal regulation that controls cochlear development and gene expression networks will provide a theoretical basis for the development of effective treatments. In addition, a multidisciplinary approach that integrates basic scientific research, engineering innovation, and clinical expertise is essential for auditory loop reconstruction.

In conclusion, future research should prioritize the development of innovative strategies to improve the survival and function of implanted stem cells and enhance the biocompatibility of materials and culture systems. With the advances in basic scientific research, engineering innovation, and clinical expertise, tissue engineering approaches have the potential to revolutionize the field of hearing repair and offer new hope for patients' hearing reconstruction.

## Conflict of Interest

The authors declare no conflict of interest.
